# Rauniticine-*allo*-Oxindole B and Rauniticinic-*allo* Acid B, New Heteroyohimbine-Type Oxindole Alkaloids from the Stems of Malaysian *Uncaria longiflora* var. *pteropoda*

**DOI:** 10.3390/molecules16086541

**Published:** 2011-08-04

**Authors:** Fatimah Salim, Nor Hadiani Ismail, Khalijah Awang, Rohaya Ahmad

**Affiliations:** 1 Faculty of Applied Sciences, Universiti Teknologi MARA, 40450 Shah Alam, Selangor, Malaysia; Email: fatimahsalim10@gmail.com (F.S.); norhadiani@puncakalam.uitm.edu.my (N.H.I.); 2 Department of Chemistry, Universiti Malaya, Kuala Lumpur 50603, Malaysia; Email: khalijah@um.edu.my (K.A.)

**Keywords:** *Uncaria longiflora*, rubiaceae, oxindole alkaloids, heteroyohimbine, rauniticine-*allo*-oxindole B, rauniticinic-*allo* acid B

## Abstract

Two new heteroyohimbine-type oxindole alkaloids, rauniticine-*allo*-oxindole B and rauniticinic-*allo* acid B, have been successfully isolated from the stems extract of Malaysian *Uncaria longiflora* var. *pteropoda*. The structures of the two new alkaloids were determined by spectroscopic analysis.

## 1. Introduction

*Uncaria* (family Rubiaceae) is a genus of woody climber plants comprising 34 species. The plants of the genus are mainly distributed in tropical regions, including Southeast Asia, Africa and Southeast America. Of these, about 10 species of *Uncaria* are found in Malaysia and *Uncaria longiflora* var. *pteropoda* is one of the representatives [1,2]. Traditionally, the leaves of *Uncaria longiflora* var. *pteropoda* are rubbed on the body to relieve pain from rheumatism, while its juice is used for thrush and mixed with iron-rust for framboesia [1]. Early chemical investigations on this plant have led to the isolation of two heteroyohimbine-type oxindole alkaloids, namely, pteropodine and isopteropodine [3,4,5]. Our recent study [6,7] has found that the plant demonstrates strong antioxidant, antimicrobial and antidiabetic potential. This prompted us to reinvestigate its chemical constituents which has now led to the successful isolation of three heteroyohimbine-type oxindole alkaloids, namely uncarine F, speciophylline and isopteropodic acid along with those previously been reported [8,9]. A further phytochemical study on the plant has afforded two new heteroyohimbine-type oxindole alkaloids, named rauniticine-*allo*-oxindole B and rauniticinic-*allo* acid B. In this paper, the isolation, structure elucidation and characterization of these two alkaloids are discussed. 

## 2. Results and Discussion

Two new heteroyohimbine-type oxindole alkaloids were successfully isolated from the methanol stems extract of Malaysian *Uncaria longiflora* var. *pteropoda*. Complete and unambiguous ^1^H- and ^13^C-NMR assignments for the alkaloids **1** and **2** were possible with the use of ^1^H-H correlated spectroscopy (COSY), heteronuclear multiple quantum coherence experiment via direct coupling (HMQC) and heteronuclear multiple bond correlation spectrum (HMBC). The DEPT experiment was used to ascertain the number of sp, sp^2^, sp^3^, and quaternary carbon atoms. Structures were also further confirmed by Fourier Transform Infra-Red Spectrometry (FTIR), Ultraviolet-Visible Spectroscopy (UV-Vis) and Mass Spectrometry (MS), as well as comparison with literature values.

**Figure 1:**
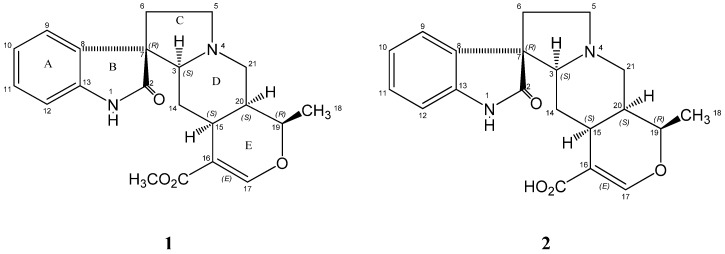
Structures of alkaloids **1** and **2**.

Alkaloid **1** was isolated as whitish colourless needles and showed a molecular ion peak at *m/z* 369.1821 [M+H]^+^ (calcd. 368.1553) corresponding to the molecular formula of C_21_H_24_N_2_O_4_. The UV spectrum showed an absorption at 238 nm indicative of an oxindole chromophore. The IR spectrum showed characteristic absorptions of a heteroyohimbine-type oxindole alkaloid observed at 3245 cm^−1^ (NH), 3120 (C-H aromatic), 1720 cm^−1^ (C=O conjugated ester), 1678 cm^−1^ (C=O amide), 1621 cm^−1^ (C=C olefinic), 1473 (C=C aromatic) and 1189 cm^−1^ (C-O cyclic ether) [4]. The ^1^H-NMR spectra displayed four aromatic protons and an olefinic signal assembled in its low field region at δ 6.87 (1H, *d*, *J* = 7.5 Hz, H-12), δ 7.08 (1H, *ddd*, *J* = 7.5, 7.5, 0.9 Hz, H-10), δ 7.20 (1H, *ddd*, *J* = 7.5, 7.5, 1.2 Hz, H-11), δ 7.22 (1H, *d*, *J* = 7.5 Hz, H-9), and δ 7.65 correlating with signals at δ 110.37, 122.75, 127.90, 122.96 and 157.25, in the ^13^C-NMR spectra, as determined by the HMQC experiment. These signals are representative of an oxindole nucleus with an unsubstituted ring A and indicate the presence of an olefinic proton at position C-17 in ring E (See Figure 1). The HMQC experiment revealed that there were four pairs of signals for non-equivalent methylene geminal protons, namely, δ 1.59 and 1.78, δ 2.41 and 3.32, δ 2.44 and 3.27, and δ 2.00 and 2.41. In the COSY spectrum the first pair showed connectivity with the signal at δ 2.34 (1H, *dd*, *J* = 12, 3.6 Hz) which is correlated with the carbon signal at δ 74.04 in the HMQC experiment. Therefore, the proton at δ 2.34 had to be assigned as H-3 since this was the only position at which a proton could have coupling with an adjacent methylene group (C-14). One of the methylene protons at C-14 showed a slight upfield shift at δ 1.59 and was assigned as 14β-H from the observation of its large coupling constant with H-15 (*J* = 12 Hz) and with H-3 (*J* = 12 Hz). The geminal pair at δ 2.41 and 3.32 (1H, *ddd*, *J* = 13, 9, 2 Hz) was correlated with the carbon signal at δ 55.31 while the geminal pairs at δ 2.44 (1H, *dd*, *J* = 12, 4 Hz) and 3.27 (1H, *d*, *J* = 12 Hz), and at δ 2.00 and 2.41 correlated with the carbon signals at δ 53.73 and δ 35.58, respectively, in the HMQC spectrum. Therefore, the second, third and fourth pairs of methylene protons were assigned at position C-5, C-21 and C-6, respectively. The coupling constant of 4 Hz between the proton at δ 2.44 and H-20 is typical of axial-equatorial coupling. The signal at δ 2.44 was thus assigned as α-oriented proton at the C-21 position. The carbonyl carbon at δ 183.06 was assigned at the C-2 position based on its long range correlation with H-6β and H-3. The other carbonyl carbon at δ 172.02 showed connectivity with H-17 and OCH_3 _and was subsequently assigned as ester carbonyl. The long range coupling observed between H-6 and δ 134.06 enabled the distinction between C-8 and C-13 quaternary carbons. The correlation shown between protons of C-5 and C-6 with the quaternary carbon at δ 56.34 confirmed its position at C-7 (See Figure 1). The relatively low field 18-H_3_ signal at δ 1.44 (*J* = 6 Hz), alkaloid **1** represents an *allo*-type isomer due to the close proximity of the 18-H_3_ to N_b_ lone pair electrons [10,11]. Two *allo*-type streoisomers are possible for alkaloid **1**; rauniticine-*allo*-oxindole A and rauniticine-*allo*-oxindole B, since the NMR characteristics of pteropodine and isopteropodine had been well established [4,8,10]. Rauniticine-*allo*-oxindole A (rauniticine oxindole A) has been reported as a constituent of Thai *Uncaria elliptica* leaves [12]. The coupling constant of 5.7 Hz (*cis*) between H-19 and H-20 in alkaloid **1** suggested the 18-H_3_ is β-oriented, thus supporting a *cis* D/E junction, comparable to a value of 6 Hz reported for rauniticine oxindole A [12]. The 14β-H signal appears at a relatively high field at δ 1.59 due to the shielding effect of the benzene ring [10] and, hence, it can be deduced that alkaloid **1** possesses the 7*R* configuration. This configuration is further supported by the slightly upfield ^13^C-NMR chemical shifts of C-14 and C-15 at δ 29.61 and δ 30.92, respectively. The stereochemistry of **1** at various stereogenic centres was assigned by close comparison of chemical shifts as reported for different stereoisomers by Seki *et al* [10]. On the basis of spectral data and comparison with literature, structure **1** was postulated as rauniticine-*allo*-oxindole B, a new naturally isolated *allo*-type isomer having 3*S*, 7*R*, 15*S*, 19*R*, 20*S* configuration.

Alkaloid **2 **was also isolated as whitish colourless needles showing a molecular ion peak at *m/z* 355.1656 [M+H]^+^ (calcd. 354.1356), 14 mass units less than that of alkaloid **1**. The molecular formula of alkaloid **2** was deduced as C_20_H_22_N_2_O_4_ on the basis of MS and NMR data. The UV region showed absorptions at 213 nm and 242 nm suggesting that a hydroxyl substituent may be present. The IR spectrum revealed the presence of non-hydrogen bonded OH (sharp peak) at 3436 cm^−1^. As in alkaloid **1** typical IR characteristics of heteroyohimbine-type oxindole alkaloids were observed at 3239 cm^−1^ (NH), 3114 (C-H aromatic), 1715 cm^−1^ (C=O acid), 1699 cm^−1^ (C=O amide), 1621 cm^−1^ (C=C olefinic), 1473 (C=C aromatic) and 1191 cm^−1^ (C-O cyclic ether) suggesting that alkaloid **2 **also belongs to pentacyclic oxindole alkaloid [4]. Except for a few signals the ^1^H- and ^13^C-NMR spectrum of alkaloid **2** bear a very close resemblance to that of alkaloid **1**. Signals for rings A, B, C, D, and E did not show any significant shift in the absorption positions in the spectra of both alkaloids. The most notable difference between alkaloid **2** and **1** was the lack of the methoxy proton and a carbon signal at δ 3.51 and δ 50.87, respectively, in alkaloid **2**. Furthermore, the presence of the carboxylic acid moiety at C-16 was confirmed by the fragment ion at *m/z* 337 and a base peak at *m/z* 209 [M-45]^+^. The stereochemistry at various stereogenic centres was assigned in a similar manner as for alkaloid **1**. Alkaloid **2** is determined to be a 16-carboxy derivative of alkaloid **1** and named as rauniticinic-*allo* acid B with a 3*S*, 7*R*, 15*S*, 19*R*, 20*S* configuration.

The occurrence of heteroyohimbine-type oxindole alkaloids containing the 18-H_3_ group with β-configuration is very rare, having only been previously reported by Phillipson and Supavita in 1983 [12]. The authors suggested that the occurrence of this type of compounds in *Uncaria* genus may be due to the transition of heteroyohimbines to oxindoles via indolenine intermediates brought about by differences in cell pH values. This was supported by their earlier study [13] on the interconversions of oxindole alkaloids whose proportions in equilibrated mixtures depend on whether they have been treated with acid or base. These findings were further confirmed by Laus *et al*. in 1996 [14]. In our investigation, the isolation of the two alkaloids from the stems, and not the leaves of the plant, despite being subjected to the same extraction and isolation procedures, ascertains that they are indeed natural products and not artifacts. 

## 3. Experimental

### 3.1. General

TLC and PTLC were performed using pre-coated aluminium-backed supported silica gel 60 F_254_ (0.2 mm thickness) and glass supported silica gel 60 F_254_ (0.5 and 1.0 mm thickness), respectively. Column chromatography was carried out using silica gel 60, 70-230 mesh ASTM (Merck 7734) whereas radial chromatography was done using glass plates with Merck’s silica gel Kieselgel 60 PF_254_ Merck Art 7749. Spots and bands for compounds on TLC, PTLC and radial plates were detected using UV light (254 and 365 nm). Mass spectra were measured on an Agilent Technologies 6224 TOF LC/MS equipped with an Agilent Technologies LC system 1200 series. The ultraviolet (UV) spectra were obtained in methanol on a Shimadzu UV-Vis 160i. The infrared (IR) data was recorded on a Perkin Elmer model FT-IR spectrometer as KBr disks. Optical rotations were measured on a JASCO P1020 digital polarimeter. Melting points were determined using X-4 melting-point apparatus with microscope JM628 digital thermometer. The ^1^H- and ^13^C-NMR were analyzed in chloroform-*D* on Bruker 300 Ultrashield NMR spectrometer measured at 300 and 75 MHz, respectively

### 3.2. Plant Materials

Plant materials were collected from Hutan Simpan Bangi, Selangor, Malaysia. The voucher specimens (HTBP 1336) were deposited at Taman Botani Putrajaya, Malaysia.

### 3.3. Extraction of Crude Extracts

The stems of plant materials were separately cut into small pieces, air-dried and ground into fine powder. The finely ground plant materials were weighed, and extracted exhaustively with methanol at room temperature for 72 hours. The solvent were evaporated off under reduced pressure and the weight of the extract was recorded. The extract was stored at 4 °C until they are subjected to acid-base extraction.

### 3.4. Acid/Base Extraction

Crude extract (25 g) was acidified with 5% HCl. Filtration to remove non-alkaloidal material followed by basification with 37% NH_4_OH released the alkaloids, which were taken up in CHCl_3_ to give a 2.25 g crude alkaloid fraction.

### 3.5. Isolation and Purification of Alkaloids

The crude alkaloid fraction (2.25 g) was dissolved in methanol and subjected to radial chromatography (4 mm thickness silica gel plate) with dichloromethane (DCM):ethyl acetate (EtOAc) followed by EtOAc:methanol (MeOH) with a gradual increase of solvent polarity to give 37 fractions. Fractions 1 and 2 were combined and subjected to PTLC in multiple developments using a 1:1 DCM:EtOAc solvent system to yield isopteropodine and pteropodine [8]. Fractions 3–6 were combined and also subjected to PTLC in multiple developments with a 4:1 DCM:EtOAc solvent system to give uncarine F and speciophylline [8,9]. Similarly, the combined fractions 7–12 was subjected to PTLC using a 24:1 CHCl_3_:MeOH solvent system to yield isopteropodic acid [8] and a mixture of **1** and **2** which was separated and purified by repeated PTLC using the same solvent system. 

*Rauniticine-allo-oxindole B* (**1**). Whitish colourless needles, mp 253–256°. [α]_D_ –58 (EtOH; *c*0.008); MS *m/z* = 369.1821 [M+H]^+^, C_21_H_24_N_2_O_4_; UV (MeOH) λ_max_ nm: 238; IR (KBr) υ_max_ cm^−1^: 3245, 3120, 1720, 1678, 1621, 1473, 1189; ^1^H-NMR and ^13^C-NMR data, see Table 1 and Table 2.

*Rauniticinic-allo acid B* (**2**). whitish colourless needles, mp 251–252° (dec.). [α]_D_ –42 (EtOH; *c*0.010); MS *m/z* = 355.1656 [M+H]^+^, C_20_H_22_N_2_O_4_; UV (MeOH) λ_max_ nm: 242, 213; IR (KBr) υ_max_ cm^−1^: 3436, 3239, 3114, 1715, 1699, 1621, 1473, 1191; ^1^H-NMR and ^13^C-NMR data, see Table 1 and Table 2.

**Table 1:** ^1^H-NMR shifts in δ for alkaloids **1** and **2** (in CDCl_3_).

H	1	2
3	2.34, *dd*, *J* = 12, 3.6 Hz	2.34, *dd*, *J* = 12, 3.6 Hz
5α	2.41, *m*	2.41, *m*
5β	3.32, *ddd*, *J* = 13, 9, 2 Hz	3.32, *ddd*, *J* = 13, 9, 2 Hz
6α	2.00, *m*	2.00, *m*
6β	2.41, *m*	2.41, *m*
9	7.22, *d*, *J* = 7.5 Hz	7.22, *d*, *J* = 7.5 Hz
10	7.08, *ddd*, *J* = 7.5, 7.5, 0.9 Hz	7.08, *ddd*, *J* = 7.5, 7.5, 0.9 Hz
11	7.20, *ddd*, *J* = 7.5, 7.5, 1.2 Hz	7.21, *ddd*, *J* = 7.5, 7.5, 1.2 Hz
12	6.87, *d*, *J* = 7.5	6.87, *d*, *J* = 7.5
14α	1.78, *ddd*, *J* = 12, 4.8, 3.6 Hz	1.78, *ddd*, *J* = 12, 4.8, 3.6 Hz
14β	1.59, *ddd*, *J* = 12, 12, 12 Hz	1.59, *ddd*, *J* = 12, 12, 12 Hz
15	2.48, *m*	2.48, *m*
17	7.65, *s*	7.65, *s*
18	1.44, *d*, *J* = 6 Hz	1.44, *d*, *J* = 6 Hz
19	4.58, *q*, *J* = 6, 5.7 Hz	4.59, *q*, *J* = 6, 5.8 Hz
20	1.65, *br m*	1.65, *br m*
21α	2.44, *dd*, *J* = 12, 4 Hz	2.44, *dd*, *J* = 12, 4 Hz
21β	3.27, *d*, *J* = 12 Hz	3.27, *d*, *J* = 12 Hz
23	3.51, *s*	*–*
NH	9.98, *br s*	10.03, *br s*

**Table 2:** ^13^C-NMR shifts in δ for alkaloids **1** and **2** (in CDCl_3_).

Carbon No.	1	2
2	183.06	183.13
3	74.04	74.04
5	55.31	55.31
6	35.58	35.59
7	56.34	56.34
8	134.06	134.08
9	122.96	122.96
10	122.75	122.74
11	127.90	127.89
12	110.37	110.40
13	140.74	140.75
14	29.61	29.61
15	30.92	30.92
16	108.69	108.72
17	157.25	157.25
18	18.95	18.98
19	72.47	72.37
20	37.86	37.86
21	53.73	53.74
22	172.02	22
23	50.87	–

## 4. Conclusions

Further phytochemical studies on the methanol stems extract of Malaysian *Uncaria longiflora* var. *pteropoda* have successfully afforded two new heteroyohimbine-type oxindole alkaloids, named as rauniticine-*allo*-oxindole B and rauniticinic-*allo* acid B. 
